# Impact of Reduced Net Height and Ball Speed Feedback on Spiking Ability in Introductory Volleyball

**DOI:** 10.3390/sports13100347

**Published:** 2025-10-03

**Authors:** M. Perla Moreno, Enrique Ortega-Toro, Alicia Lara, Aurelio Ureña

**Affiliations:** 1 Department of Physical and Sports Education, Faculty of Sports Sciences, Sport and Health University Research Institute (iMUDS), University of Granada, 18011 Granada, Spain; perlamoreno@ugr.es (M.P.M.); aurena@ugr.es (A.U.); 2 Faculty of Sports Sciences, University of Murcia, 30107 Murcia, Spain; 3 Sport Performance Analysis Association, 30720 Murcia, Spain; 4 Master in Physical Activity & Sport Research, Department of Physical and Sports Education, University of Granada, 18011 Granada, Spain; alicialara@correo.ugr.es

**Keywords:** constraints learning approach, sports initiation, attack, vertical jump height, spike velocity

## Abstract

The aim of the study was to explore the impact of an intervention based on the manipulation of the margin of error and the provision of ball speed feedback on the ability to spike in introductory volleyball. To this end, an exploratory study without a control group was conducted. The sample consisted of two U-14 volleyball teams, one male team with 14 players (13.2 ± 0.75 years), and one female team with 12 players (14 ± 0 years). The intervention involved reducing the height of the net, providing immediate feedback on the speed of the ball after the spike, and challenging the target zone of the spike. It was applied across 12 sessions, with eight spikes per player per session. The study variables recorded in each spiking were ball speed (which was measured using the Pocket Radar Ball Coach instrument), jump height (which was measured using the VERT Wearable Jump Monitor), and target area for sending the ball (which was filmed using a high-speed video camera). The players’ perception of the intervention was also assessed. The most significant results indicated that the achievement of the impact in the more restricted target area of the spiking, compared to the larger target area, led to a significant increase in jumping, both in men and women. As maintaining spike ball speed was necessary to validate the challenge, speed values did not decrease when hitting toward the restricted zone. In fact, for male players, there was an unexpected significant increase in spike ball speed. The initial speed was the variable that best predicted the maximum speed acquired throughout the treatment. Reducing the net height while restricting the spiking area can have a positive impact on spike kinematics, provided that spike velocity is maintained.

## 1. Introduction

For many years, the teaching of volleyball in sports initiation stages has been marked by the search for modifications of the game that would allow it to be adapted to the characteristics of the players. These proposals for the initiation of volleyball, using adapted, simplified, modified games, usually called small-sided games, have usually been governed by increasing the number of contacts [1], with the underlying priority of promoting ball control and precision [2]. However, recently, trends with a different direction are emerging from the practice itself in the initiation of volleyball. Examples such as Smashball [3] and Monster Block Teen’s [4], which emphasize actions played at the net, propose a reduction in net height. Moreover, currently, from the Dual Convergent Model for volleyball initiation [2], it is considered that in the different volleyball game actions, different primary motor objectives predominate (speed or precision), which must be prioritized during the learning process. Actions such as spiking, blocking, or powerful serves have as their primary motor objective the search for speed. On the other hand, from various models that consider physical development to be encouraged in athletes throughout the different formative stages, it is established that, at the age of initiation to sport, it is absolutely essential to stimulate the speed of movement in children [5,6,7].

From these considerations, Ureña et al. (2022) proposed that different game actions in volleyball could be taught using tasks with specific characteristics [2]. This law establishes an inverse relationship between the speed and the precision or difficulty of the task (determined by the distance and width of the target, which represents the margin of error). In this sense, the use of different motor patterns was identified to adapt to increasing levels of difficulty in a specific Fitts task [8]. Furthermore, when not wanting to fail, or if the task reduces the error to 0%, practitioners try to reduce the trajectory variation and increase the movement time [9]. Therefore, movements with longer distances and shorter durations would result in a lower probability of hitting the target, and shorter distances and longer durations would lead to greater accuracy and hitting the target, but with slower movement time [10].

Specifically in volleyball, the margin of error can be established through an inverse relationship with the relative height of the net (depending on the player’s reach) and a direct relationship with the size of the target [2]. This idea is shared with Fuchs, Menzel et al. (2019, p. 2411), indicating that the greater the jump height achieved in the spiking, the larger the effective field size, and the steeper the trajectory of the ball at high speed [11].

The application of this assumption, based on the margin of error, is in line with the conceptual framework of the ecological perspective, dynamic systems, and the Constraints Learning Approach (CLA) [12], in which skill is considered as an evolutionary adaptation to a dynamic environment, which can be modified through constraints of the task, the organism, or the environment. Thus, the modification of the height of the net or the sending zone of the ball, in volleyball, is considered a task constraint and would enable the achievement of specific goals through implicit learning. Maxwell et al. (2000) refer to implicit motor learning as ‘the acquisition of a motor skill without the simultaneous acquisition of explicit knowledge about the performance of that skill’ [13].

Furthermore, one way to promote implicit, rather than explicit, learning is by orienting to an external attentional focus. As Wulf et al. (2001) indicated, as a result of their review and study on the subject, the attentional focus toward which learners are directed, induced by the input of instructions or feedback, has a significant impact on the learning of motor skills [14]. Among other circuits, a distinction is made between an internal and an external attentional focus, respectively understood as attention to body movements, or attention to the effect of movements on the apparatus or implement, i.e., on the medium [14]. Results from previous studies in different sports and skills have shown the advantages of inducing an external rather than an internal attentional focus (i.e., feedback toward an external attentional focus in volleyball serving resulted in higher accuracy of serves during practice and retention) [15]. Furthermore, this approach is shown to be most effective in motor tasks where speed is critical to improve performance in the countermovement jump in baseball [16,17]. Specifically, in volleyball spiking, studies found that providing instruction with an external rather than an internal attentional focus or using unfocused instruction in volleyball players with a mean age of 13.6 years produced significant differences in hand, forearm, and arm speed increases in spiking from the beginning of the wind-up phase to the acceleration phase prior to contact with the ball [18].

Based on this background, the idea of lowering the height of the net to improve, fundamentally, the speed of the ball after the spiking may be promising [2].

However, there are some objections to this expectation. The main one is that focusing on the challenge of achieving maximum speed, with a lower height requirement, may be detrimental to the vertical jump height applied to the spike in that situation. To mitigate this effect, a secondary challenge could be proposed that would consist of searching for a target zone closer to the net, as long as the speed values are preserved. This is because the height of the spike determines the possible angle of incidence of the spike. This solution to the first problem raises a second objection, which is the possible conflict between a task constraint that widens the margin of error in the vertical plane and a second constraint that reduces the margin of error in the horizontal plane.

This alternative approach to volleyball initiation has not been contrasted with research results, so it is considered necessary to carry out exploratory studies on the subject, which, through the exploration or description of the data, allow us to generate hypotheses that can be considered in future confirmatory studies [19].

Therefore, the general objective of the present study was to explore the impact of an intervention based on reducing the height of the net, providing feedback on the speed of the ball after the spike, and incorporating a challenge with respect to the target zone for sending the spiked ball, which could be affected by the height of the jump, on the ability to spike (ball speed after the spike and vertical jump height during the spike).

## 2. Materials and Methods

### 2.1. Design and Sample

An exploratory study was conducted in two Under-14 (U-14) volleyball teams (male and female). This is a mixed methods study (quantitative and qualitative) of recognized usefulness and value in the field of physical activity and sport [20].

The sample, obtained through non-probabilistic, convenience sampling, consisted of 26 U-14 volleyball players, belonging to the male category (*n* = 14; M = 13.2; SD = 0.75), and to the female category (*n* = 12; M = 13.82; SD = 0.39). The legal guardians of the players signed an informed consent form in which they accepted and committed to their participation in the study (this study was performed in line with the principles of the Declaration of Helsinki. Approval was granted by the Ethics Committee of the University of Murcia: 4733/2023). On behalf of all authors, the corresponding author states that there are no conflicts of interest.

The criteria for the inclusion of the players in the study were as follows:

- Membership of one of the study teams; signing of the informed consent form by the legal guardians.
- Attendance to at least 75% of the intervention sessions; recording speed in at least 33% of the total number of spiking actions carried out; response to the semi-structured interview.

### 2.2. Intervention

The intervention applied during spiking training was based on the conceptual framework of the CLA [12]. The specific learning objective takes into account recent practical trends in volleyball initiation regarding spiking. Other theoretical considerations supporting the advantages of the CLA for this study include the pursuit of an optimal challenge [20] and the benefits for motor learning of directing attention toward an external focus [18].

Accordingly, the intervention in the spike was characterized by (a) reducing the height of the net, maintaining a wide target zone; (b) inducing the attention of the players toward an external attentional focus, through the incidence in the instructions on the objective of seeking maximum speed in the spike, and the use of augmented extrinsic feedback, consisting of the immediate indication after the completion of the spike of the achieved value of the ball speed; (c) to stimulate those players who managed to establish high values of speed in the spike to try to send the spiked ball to an area closer to the net, which could be affected by the height of the jump. Specifically, the net height for the U-14 boys was 2.13 m (5% lower than the regulation height, i.e., 11.2 cm less), and for the U-14 girls it was 2 m (5% lower than the regulation height, i.e., 10.5 cm less). Initially, the players had the whole court, a large area of (9 × 9 m), to send the ball in the spike. Only in the case that a player achieved 4 spikes in a session that exceeded 90% of the maximum speed of the ball in his/her spiking (maximum value registered in the first session of intervention or in any of the following sessions), they were challenged to try to maintain high speed values in the following session by sending the ball to a restricted area, specifically to the transversal half of the opponent’s court closest to the net (9 × 4.5 m). This choice of court areas was aimed at promoting higher spikes without significantly reducing the margin of error. After each spiking, the player was immediately informed about the speed of the ball after his/her spiking when the ball was sent to the cross-court half of the opponent’s court closest to the net.

In order to give immediate feedback on the progress of the individual challenge of sending the spike ball to a restricted area, if applicable, all spikes were recorded in an Excel 2024 sheet, recording the maximum spike ball speed achieved by each player (which would be updated during the intervention), as well as the ball speed after the spiking and the destination zone of the ball, for each spike. In this way, it was possible to know and indicate the situation of each player at each moment, and if he/she should advance in the challenge. The intervention on the spiking was applied during a total of 12 training sessions (six weeks), in two of the team’s three weekly training sessions, and with a duration of 45 min per session. In each intervention session, each player performed eight spikes of a ball thrown from the dominant hitting zone (Zone 4 or 2). In the absence of other experimental precedents related to the issue, this number of spikes per player per training session was established to make reasonable use of the actual training time available to the team.

### 2.3. Study Variables

During the study, we recorded variables of the spiking ability that, in previous research, have been shown to be relevant to spiking success, such as ball speed after spiking [21,22] and the height of the vertical jump in spiking [22]. Specifically, the study variables considered were the speed of the ball after the spike, the height of the vertical jump in the spike, the target area where the ball impacts, and the number of sessions. The players’ perceptions of the intervention were also analyzed.

- Ball speed after the spiking is understood as the maximum speed, in km/h, that the ball reaches after the player hits the shot. From this variable, the following was calculated for each player:
  - Initial speed—understood as the median value of the ball speed after the spike in the first session;
  - Maximum speed reached—understood as the highest median ball speed after the spike among the 12 sessions;
  - Session with the maximum speed—understood as the ordinal of the session in which the maximum value of ball speed after the spike was reached;
  - Maximum increase in speed—understood as the proportion of the variation in ball speed after the spike between the session with the highest value and the initial session.
- Vertical jump height in the spike is understood as the maximum vertical height, in centimeters, that the player reaches during the flight phase of the spike jump. Based on this variable, the following was calculated for each player:
  - Initial height—understood as the median value of the vertical jump height in the first session;
  - Maximum height reached—the highest median vertical jump height of the 12 sessions;
  - Session with the maximum height—understood as the ordinal of the session in which the maximum value of the vertical jump back-top height was reached;
  - Maximum increase in height—understood as the proportion of the variation in the vertical jump back–top height between the session with the highest value and the initial session.
- Target area is understood as a specific area of the opponent’s court to which the spiking is directed. Categories were differentiated as extensive, when the ball was sent to any area within the 9 × 9 m of the opponent’s court; restricted, when the ball was sent to the transversal half of the opponent’s court closest to the net (9 × 4.5 m); and off-target, when the spiking hit outside the regulation delimitation.
- Participation in the program is understood as the number of sessions in which each player participated. In addition, the players’ perception of the intervention was considered, once the intervention had concluded, through the analysis of the response to a semi-structured interview.

### 2.4. Measurement and Instruments

The Pocket Radar Ball Coach instrument (Pocket Radar, Inc., Santa Rosa, CA, USA), which measures the speed of a ball over a range of 35 m, was used to record the speed of the ball after the spiking. The radar has been validated as a device for recording spike velocity, showing a correlation of 0.98 with measurements obtained through photogrammetric analysis [23]. Two such devices were used in this study and were placed on the opposite side of the field to where the players were shooting, each on the side of the back line of the field. Two devices were used to ensure that, regardless of the area of the field to which the spiking was directed, ball speed data were always collected after the shot. The velocity value recorded by the device closest to the direction of the spike was considered. Similar protocols to the one employed in the present study, but using a single device and establishing the spike direction, have been used in research on high-level volleyball to measure spike velocity in a natural context [22,24].

The players’ vertical jump height was recorded using the VERT Wearable Jump Monitor (Mayfonk Athletic, LLC, Fort Lauderdale, FL, USA), a system that provides real-time information using an inertial measurement unit (IMU), the size of a USB stick, which is attached to a belt. Previous research on the validation and use of this tool in volleyball has determined that, in a simple manner and without requiring time-consuming analysis or custom software, it has the potential to measure a vertical jump in volleyball, as correlations with three-dimensional motion analysis yielded values of r = 0.83–0.97 [25]. It provides an acceptable measure of jump height on the court, although its use is not recommended when high precision is required [26], it is valid and precise for quantifying performance in attack and block jumps in volleyball on the court, with acceptable validity and precision for use in training and competition [27], and it has been employed in studies analyzing jump height in volleyball [28]. Considering the advantages and limitations of using the device, for a non-invasive ecological context and given the aims of this study, its use appears to be a suitable choice. This device features wireless communication that synchronizes data into an Excel file. To relate the recording to the corresponding action, the time recorded by the VERT Wearable Jump Monitor was linked to the camera recordings for further analysis.

The target area of the ball delivery was measured by systematic observation. This observation was carried out by an observer after a process of observer training in which the consensus and reliability of the observation were guaranteed (intra-observer Cohen’s Kappa greater than 0.75).

The recording of the spikes developed during the intervention was carried out by placing a Sony CX625 model camera on the side of the court opposite where the spike was taken, specifically in the 3-meter zone near the net, with a separation of 2 m from the sideline of the court.

An Excel spreadsheet was designed to record the data for the three variables indicated above, which allowed the collection of information on the speed of the ball after the spiking (provided by the Pocket Radar Ball Coach instrument), the target area for sending the ball in the spiking, and the number of attempts made by each player. In addition, this system allowed us to subsequently link the vertical jump height (provided by the VERT Wearable Jump Monitor) with each of these attempts.

To find out the players’ perception of the intervention applied, a semi-structured interview of 4 questions was used, to which the players responded once the intervention was over.

1. Do you think that this way of training the spike has made you increase your jump?
2. Has it been a stimulus for you to learn about spike speed? In what way?
3. Did you learn how to calculate your spike speed before you were told the radar data? From when do you think you were able to calculate the speed?
4. Do you think that training with the net at a lower height than the regulation one helped you to improve your level of play during the competition matches? Why?

After the transcription of the interviews, the thematic analysis of the interviews was conducted [29,30].

### 2.5. Statistical Analysis

The nature of the exploration design employed guided researchers to reinforce the results with the athletes’ own perception of the intervention applied, carrying out a mixed-methods study. Accordingly, quantitative data analysis was carried out (presented below), as well as qualitative thematic analysis of the players’ perceptions.

After an initial comparison between male and female categories, these were treated in a segregated manner. The data were organized for analysis in two ways. On the one hand, an analysis was made based on the successive records of each spiking, looking for relationships between speed, height, and the area where the ball was hit. The Kolmogorov–Smirnov normality test was applied for the speed and height variables. The result was non-normal for both variables. In addition, the speed variable presented such a distribution that it could not be corrected after applying several transformation methods. Therefore, Spearman’s coefficient was used for the correlation analysis of both variables. The Kruskal–Wallis test for independent samples was used to establish the differences in the distribution of speed and height depending on the sending areas of the backswing, and for the pairwise comparison of the three response levels, the significance adjustment after Bonferroni correction was taken into consideration.

For the second orientation of the analyses, a representative value of the ball speed and jump height achieved was attributed to each player in each session. Given the high variability of the individual data in only eight runs per session, it was decided to take the median of both variables for each player and session. This approach sacrifices the ability to detect specific trends in each session in order to track the individual progression of each player throughout the intervention. As second-order variables, the initial value and maximum value reached were calculated, together with the number of sessions in which this maximum occurred and the percentage increase of the maximum value with respect to the value of the initial session. All of these were performed both for the ball speed after the spike and for the vertical jump height during the spike. After applying the Shapiro–Wilk normality test (samples of 14 and 12 players, respectively), some of the variables met, and some did not, the assumption of normality. For relationships involving non-normally distributed variables, Spearman’s correlation coefficient was applied, and for relationships involving exclusively normally distributed variables, bivariate correlations were described with Pearson’s coefficient, and simple and multiple linear regression analyses were applied.

## 3. Results

The following results are presented: the difference and relationship between the vertical jump height in spiking and ball speed after spiking; the effect of target area restriction on ball speed after spiking and vertical jump height in spiking; the effect on vertical jump height in spiking; and participation in the program. In addition, a thematic analysis of player perception is included.

- Difference and relationship between vertical jump height and ball speed after the spiking as a function of the category.

The Mann–Whitney U-test indicated that the distribution of speed and height was significantly different for both categories (*p* = 0.001). For height, the average range for the male category was 952.82, compared to 630.16 for the female category. For stroke speed, the male category had an average range of 918.86, compared to 641.05 for the female category. For both categories, there was a moderate correlation between the ball speed after the spiking and jump height. For the male category, it was ρ = 0.534, *p* < 0.001; while for the female category, it was ρ = 0.556, *p* < 0.001.

- Effect of target area restriction on ball speed and vertical jump height in spiking.

Pearson’s chi-square test yielded a significant difference (*p* < 0.001) between categories, albeit with a small Cramer’s V value (0.143). Corrected residuals showed significant differences between the large area (females: 79.9%; males: 69.3%; with a residual of +5.3) and the off-target area (females: 12.6%; males: 23.7%; with a residual of +5.7). The restricted area was 8% in the male category compared to 7.5% in the female category, with the residuals not explaining a representative difference in this area.

For the male category, the Kruskal–Wallis test showed that the target area conditioner significantly differentiated the distribution of speed (*p* = 0.001; η^2^ = 0.013) and height (*p* = 0.007; η^2^ = 0.009). The restricted area presented higher values than the large area in the male category (Table 1). These were significant, according to the Bonferroni adjustment, for both height (*p* = 0.005) and speed (*p* = 0.004). For the female category, the Kruskal–Wallis test indicated that the target area condition did not affect the distribution of the spike speed (*p* = 0.064). However, the spike jump height was significantly different as a function of area (*p* = 0.002; η^2^ = 0.015). Height values were significantly higher in the restricted area than in the large area (adjusted sig. = 0.001).

### 3.1. Effect on Vertical Jump Height in Spike

To reach the session with the maximum height, the male category needed a mean of 7.2 sessions and a median of 7.5, out of the 12 possible sessions, with a standard deviation of 3.06. For the female category, a mean of 5.4 and a median of 5.5 sessions were needed, out of the 12 possible sessions, with a standard deviation of 3.96. The increase in height between the initial session and the session with the highest value was 16.64% mean, with a median of 12.5% and a standard deviation of 16.67 for the male category, while for the female category, the increase in height was 11% mean, with a median of 9% and a standard deviation of 11. The two distributions met the Shapiro–Wilk assumption of normality in both categories. For the male category, the value of the session with the maximum height achieved was strongly correlated with the height of the first session (r = 0.714; *p* = 0.002) and with the maximum height increase achieved (r = 0.563; *p* = 0.018). A multiple linear regression model using the input method showed that the maximum height attained was explained by the height obtained in the first session and the percentage of the maximum height increment attained, with an adjusted r^2^ = 0.987. The ANOVA test indicated that the model was statistically significant (*p* < 0.001). The two independent variables showed significant participation (*p* < 0.001). The value of Pearson’s correlation coefficient below 0.900 and the collinearity statistic (VIF = 1.029) for both variables support the principle of non-collinearity. For the female category, the maximum achieved height increase showed a strong inverse correlation with the height of the first session (r = −0.787; *p* = 0.001) and a strong direct correlation with the session in which this maximum value was reached (r = 0.681; *p* = 0.007). A multiple linear regression model using the input method showed that the percentage increase in the maximum height reached is explained by the height obtained in the first session and by the maximum height reached, with an adjusted r^2^ = 0.679. The ANOVA test gives significance to the model (*p* = 0.002). Of the two independent variables, there is a significant participation in the model of the height of the first session (*p* = 0.013), but not of the session with the best height (*p* = 0.076). The value of Pearson’s correlation coefficient is below 0.900, and the collinearity statistic (VIF = 1.304) for both variables supports the principle of non-collinearity. On the other hand, in the female category, the first session height variable was also strongly correlated with the maximum height achieved (r = 0.927; *p* < 0.001), providing a coefficient of determination r^2^ = 0.860 in a significant (*p* < 0.001) simple linear regression model. Thus, for both categories, the initial height seems to determine the expectation of the maximum height reached, although the increase in height has a direct relationship with the value of that first session in the male category and an inverse relationship in the female category. These results should be interpreted with caution due to the exploratory nature of the study and the characteristics of the sample.

### 3.2. Effect on Ball Speed After the Spike

To reach the session with the highest ball speed, male participants required an average of 5.9 sessions and a median of 6.5 out of 12 sessions, with a standard deviation of 3.5. In contrast, female participants required an average of 6.4 sessions and a median of 6, with a standard deviation of 3.37. The average increase in ball speed from the initial session to the session with the highest value was 11.7% (median = 11%, SD = 7) in the male category and 7.8% (median = 7.5%, SD = 6.1) in the female category. In the male category, the variable representing the maximum ball speed did not meet the assumption of normality. This variable showed a strong correlation with the speed in the first session (ρ = 0.770; *p* = 0.001) and with the number of sessions completed (ρ = 0.619; *p* = 0.018). In the female category, the variables related to the initial and maximum ball speeds met the assumption of normality. Correlation analysis revealed significant associations between maximum speed and speed in the first session (r = 0.856; *p* < 0.001), jump height in the first session (r = 0.626; *p* = 0.029), and maximum jump height (r = 0.800; *p* = 0.002). A multiple regression model using a backward stepwise approach progressively eliminated the independent variables of the initial and maximum height (in that order). In the third step, retaining only the speed from the first session as a predictor, the model was statistically significant (*p* = 0.029), with an adjusted R^2^ = 0.707. When testing a model with the height-related variables (initial and maximum), the initial height was removed, and although the model remained significant (*p* = 0.003), its explanatory power decreased (adjusted R^2^ = 0.604). Forcing a simple model with only the initial height as the dependent variable yielded a significant result (*p* = 0.029), but with even lower explanatory power (R^2^ = 0.331). In summary, considering the results with caution due to the exploratory nature of the study and the characteristics of the sample, jump height contributed to explaining the maximum ball speed, with the maximum jump height showing greater predictive power than the initial height. However, the variable that best explained the maximum speed was the initial speed.

### 3.3. Program Participation

In the male category, the mean and median number of sessions attended was 10.5 out of a possible 12, with a standard deviation of 1.28 and a range of 3. The only variable significantly associated with the number of sessions attended was the maximum ball speed achieved (ρ = 0.619; *p* = 0.011). In the female category, the mean number of sessions attended was 10.9 out of 12, with a standard deviation of 1.28, a median of 11, and a range of 3. The only variable significantly associated with the number of sessions attended was the speed in the first session (ρ = 0.684; *p* = 0.014).

### 3.4. Thematic Analysis of Players’ Perceptions

The following is a descriptive analysis of players’ perceptions regarding the implemented intervention (Table 2).

### 3.5. Results of the Qualitative Analysis of Player Interviews

The results of the qualitative analysis of the interviews indicate that most male players (71.43%) and female players (66.67%) perceived an improvement in their jump performance because of the intervention. All participants (100%) reported that receiving immediate feedback on the ball speed after the strike served as a motivating factor for improvement. However, 33.33% of the female players (four players) and 14.29% of the male players (two players) also reported experiencing negative emotions such as sadness, anger, or frustration when they did not achieve the expected results. These sentiments are illustrated in the following excerpts (using pseudonyms): “Yes, well I didn’t like it, but it’s good to know so you can improve and all that. Because it was low and it was difficult to increase it, and that was frustrating.” (Judith); “Well yes, if it didn’t go well, of course I got angry or sad or I don’t even know what.” (Ingrid); “At first it was shocking, but then I realized I wasn’t hitting it hard, so it made me feel down.” (Soraya); “Yes, it was a surprise at first. Then, if it was low, you’d feel disappointed, and if it wasn’t, you’d be happy.” (Roberto). A total of 57.14% of male players and 50% of female players stated that they learned to estimate the approximate speed of their strike. Among those who reported acquiring this ability, the most common response in both groups was that they developed this skill early on (before the midpoint of the intervention), with 21.43% in the male category and 25% in the female category. Regarding whether players perceived an improvement in their performance during matches, 50% of the male players and 66.67% of the female players responded affirmatively. They mentioned factors such as feeling more confident when striking, hitting the ball with greater force, or directing it more downward. The following excerpts illustrate these perceptions: “Now I feel—I think I do—I feel more confident when hitting the ball, so yes, more confident when striking.” (Basilio); “Yes, as I said earlier because I’ve gained confidence regarding striking.” (Eric); “Yes, I think so. Before, my strikes would go too far, and now they stay more within the court—they used to go out more often, now they go in more.” (Nuria); “I think so, like now I hit the ball harder when I strike.” (Noemí). Meanwhile, 21.43% of male players and 25% of female players believed that their in-game performance had not improved. Additionally, one male player (7.14%) offered a nuanced perspective, explaining that during matches, he often had to prioritize control over power, leading him to feel that his level of play had “both improved and unchanged”.

## 4. Discussion

Previous approaches to manipulating space for learning specific volleyball skills are based on the application of small-sided games. Methodological proposals have mostly focused on strategies that reduce the number of players and available space [31,32]. Mahedero et al. (2015) [33], using 1v1, 2v2, 3v3, and 4v4 games, found improvements in decision-making, but a general lack of improvement in skill acquisition across three groups with different initial skill levels. In this regard, Trajkovic et al. (2017) [34] found positive outcomes for passing skills (overhead and forearm), serving, and serving under fatigue, while Pekas et al. (2019) [35] reported improved passing accuracy. When seeking conditions that enhance precision-based skills, reducing space appears beneficial. Conversely, the development of skills requiring execution speed might benefit from the opposite conditions. Additionally, recent studies have examined the effects of reducing net height below the official standard [2,36].

Spikes are a complex skill that integrates jumping ability with an approach run and a hitting action. This has been the focus of prior studies seeking to characterize performance in these two components. Fuchs, Fusco et al. (2019) [37] analyzed which factors in women’s volleyball characterized jump performance and spike ball speed. Greater jump heights have been linked to more successful attacks in youth players [38]. A significant and positive correlation has also been found between vertical jump height and spike ball speed as measured by radar [22]. In the present exploratory study, this relationship was shown to be statistically significant.

The aim of this study was to explore the influence of a training intervention—based on net height reduction, feedback on spike ball speed, and the inclusion of a target area challenge potentially affected by jump height—on spiking performance (ball speed and jump height). Properly adjusted challenges are a key element in deliberate practice [39], and “finding activities where the ever-changing challenge-skill balance tilts toward challenge provides a chance for growth, a chance to enter flow (p.63)” in pursuit of optimal performance [40]. In line with this, all players in the present study reported that immediate feedback on spike ball speed was a motivating factor for improvement, although some also reported negative emotions such as frustration, sadness, or anger when results did not meet expectations. These negative emotions were more frequent in female players than in male players, potentially reflecting a greater concern with errors among females and representing an aspect to be considered in the training process.

The primary objective of this study was to address logical concerns related to this approach. The main concern was that focusing on achieving maximum speed with lower demands on height could negatively affect vertical jump height during the spike. To mitigate this effect, a secondary challenge was proposed: aiming the spike toward a closer target zone, provided that ball speed values were maintained. However, this solution raised a second concern: a potential conflict between one task constraint that increases the vertical margin of error and another that reduces the horizontal margin. Izquierdo et al. (2008) and Ureña et al. (2022) [2,41] emphasized the need to prioritize movement learning where both speed and accuracy coexist as goals. In this study, spike speed was considered the primary objective and precision the secondary one, with the aim of maximizing the potential of the specific skill.

Sleimen-Malkoun et al. (2013) [8] identified two phases in movement execution for a discrete Fitts’ task: acceleration time (AT) and deceleration time (DT), finding variability in these phases based on the task’s index of difficulty (ID). A transition threshold in ID revealed abrupt changes in the variability of the AT/DT ratio. Similarly, Meyer et al. (1988) [42] described that fast movements toward a specific target consist of a primary movement followed by an optional secondary corrective movement. They observed that increased speed led to greater temporal variability between these submovements while maintaining overall timing and high target accuracy. However, when tasks aim for zero error, trajectory variability decreased, and movement time increased [9].

In this study, spikes aimed at the restricted target area showed significantly greater jump heights for both boys and girls. Therefore, for the task designed, constraints in spike depth required greater jump performance. As maintaining spike ball speed was necessary to validate the challenge, speed values did not decrease when hitting toward the restricted zone. In fact, for male players, there was an unexpected significant increase in spike ball speed. A notable difference between genders was that female players had half as many spikes outside the target area compared to male players. Possibly, a lower tolerance for error among male players (with less negative emotionality, such as fear or frustration in the face of potential failure) led to increased speed, even in the restricted zone. This explanation aligns with the findings of Murakami and Yamada (2021) [9].

Still, successful spikes in the restricted zone occurred in fewer than 10% of attempts in both groups. This small percentage of success in the restricted zone highlights the need to increase the margin of error, which could be addressed in two ways: either by maintaining the horizontal restriction and reducing the net height, or by keeping the net height constant and expanding the restricted zone (for example, by establishing three transversal bands instead of two).

Most players perceived an increase in their spike jump height, despite receiving no feedback on this variable. The immediate feedback on spike ball speed helped nearly half of the players learn to estimate their spike speed without many sessions. Although this was not the intervention’s primary aim, it highlights the potential value of gradually decreasing augmented feedback frequency in future studies. This could help players integrate the information into their intrinsic feedback and relate perceived spike sensations to actual performance.

Half of the male players and over half of the female players reported improved in-game performance, noting increased confidence, feeling more secure when spiking, or hitting harder and more downward.

Players’ level of involvement during training can be influenced by many factors unrelated to the task itself. However, in the male group, there was a significant direct relationship between the number of sessions and maximum speed achieved, suggesting that exposure to the intervention may have enhanced speed. In the female group, participation was significantly related to initial speed, possibly because better performance in the first session encouraged greater engagement.

Regarding the relationship between height and speed, results revealed gender-based differences. For boys, initial jump height and speed predicted maximum values achieved. For girls, spike jump height was a stronger predictor of maximum spike speed than initial jump height, but the most strongly associated with maximum spike speed was initial ball speed. In this case, the explanatory power provided by the statistical analysis may be attributable to the specific characteristics of these particular groups and should not necessarily be generalized to other groups of the same age. In terms of spike jump height increase, for boys, it was directly related to both maximum and initial jump height. For girls, the maximum increase had an inverse correlation with initial jump height.

Moreover, in the female group, the greater the jump height increase, the more sessions were needed to reach peak jump performance. That is, girls with higher initial jump height showed less improvement and reached their best performance earlier, while boys with better initial results showed greater improvement and took longer to peak. The results are promising for this male group, but the intervention seems to have had a greater impact on girls with poorer initial metrics and lower expected performance in girls with stronger initial values. Biomechanical differences in spiking between sexes have been documented. In a comparative biomechanical study of elite male and female volleyball players [11], significant gender differences were found in numerous variables, not fully explained by strength differences, but also by technical and coordinative factors. The Youth Physical Development (YPD) and Athletic Skills Model (ASM) expand upon the Long-Term Athlete Development Model (LTADM) and use Peak Height Velocity to situate physical and motor development in young athletes [5,6,43,44,45]. Although maturational age data were not collected in the present study, the observed differences in spike parameters may be related to mismatches in maturation age between boys and girls of this chronological age.

From a volleyball perspective, and especially concerning spiking, movement speed is key to understanding jump and ball speed performance. The maturation stage is also crucial in designing effective training [2,6].

Although chronological age is not definitive, it is expected that the girls in the sample (ages 12–13) had already passed their PHV, whereas the boys (ages 11–13) were likely in a pre-PHV phase. Strength training programs targeting upper body muscles have proven effective in adult female volleyball players, even during competitive phases [46]. Similarly, a plyometric program focusing on upper body strength increased spike speed by 3.8% in professional female players [47]. While strength clearly contributes to ball speed, strength training tends to yield better results post-puberty. According to Lloyd and Oliver (2012), prepubescent children benefit more from training that requires high levels of neural activation, whereas adolescents should focus more on strength training, plyometrics, and speed training to maximize overall speed gains [5]. Therefore, improvements in coordination and faster movement production by the nervous system may be more effectively developed in middle childhood [6], even before specialization in a single sport [48]. Given all of this, it cannot be ruled out that the boys increased their jumping ability, and those with higher initial values may have experienced greater gains. However, if we assume that the girls are in the post-pubertal phase, rather than expecting an increase in jump capacity, the improvement may be due to their jump ability better adapting to the technical execution. Although there is insufficient evidence to confirm this relationship, it appears consistent with improvements observed in the female players who initially exhibited lower jump levels.

Therefore, these results support the implementation of learning situations involving low net height and target zone challenges for volleyball spiking, especially in pre-pubertal stages, or later to refine coordination in players with lower kinetic performance when introduced to the sport. However, in players who have passed their PHV, strength development may have a greater impact than expected changes in movement patterns. In adolescent volleyball players, significant sex-based differences in spike jump have been found, favoring boys [49]. Nonetheless, when working with U-14 male and female players, it is essential to consider the likely motor maturation advantage of girls. Throughout the current study, however, boys consistently showed higher values in both speed and jump height. Given the sample characteristics, potential selection bias in club-level sports recruitment should also be considered.

Regarding the limitations of the study, it should be noted that the starting point is a theoretical reflection based on previous empirical contrasts that are distant from the skill and task addressed in this experiment. For reasons of caution, an exploratory study without a control group was chosen, using a non-probabilistic, convenience sample in its natural context. This limits the adequacy of sampling, both qualitatively and quantitatively. Although predictive tests could formally be applied, the results should be interpreted with caution due to the exploratory nature of the study and the characteristics of the sample.

The advantage of experimenting with a real team is that a practical intervention can be transferred to a training context. However, a team cannot even be representative of its own competition, as participant level is influenced by multiple factors, and natural inequality exists between groups. Similarly, in this natural context, establishing a control group alongside an experimental group is impractical. Nevertheless, the study design employed has allowed the integration of quantitative analysis with a qualitative approach to the phenomenon.

On the other hand, a limitation is the absence of kinematic data that could explain changes in movement patterns. For example, from a jumping perspective, it would be interesting to differentiate whether improvements reflect actual increases in jump height or better temporal adjustment that allows more effective use of available jump capacity. From a striking perspective, it would be valuable to assess whether changes occur in the contribution of segments within the kinetic chain or merely an increase in velocity for the same movement.

Additionally, measures of the players’ maturational age were not included, which would allow for a more specific interpretation. Based on these results, a larger and well-stratified sample in natural contexts using mixed designs, or laboratory studies with control group designs, including consideration of kinematic and maturational variables, appears promising.

## 5. Conclusions

Challenges hold significant potential in the development of motor skills, provided their consequences are carefully analyzed and adapted, and the coexistence of multiple simultaneous challenges within the learning task is properly structured. Moreover, performance-based challenge tasks are compatible as a complement to small-sided games within a methodology grounded in the Constraints-Led Approach. Preliminary evidence suggests that, in the learning process of spiking in introductory volleyball, reducing the net height—combined with a speed target and a restricted hitting area—could positively influence the kinematics of the spike. The potential conflict arising from responding to one constraint that increases the margin of error and another that decreases, in this sample, appears to be effectively addressed by this dual challenge (speed and height) when structured hierarchically. Participation in spike tasks involving constraint manipulation, such as those presented in this study, was considered by players as both stimulating and beneficial to their spiking performance.

## Figures and Tables

**Table 1:** Vertical jump height in spiking and ball speed after spiking, classified by target area and differentiated by category.

Target Area		Male		Female	
		High (cm)	Speed (km/h)	High (cm)	Speed (km/h)
Off-target	Mean	46.34	51.21	40.41	45.88
	Standard Deviation	10.38	9.23	7.35	5.62
	Median	46.70	50.00	40.30	43.00
	Range	55.00	44.00	35.00	23.00
	Median Deviation	8.58	7.29	5.89	4.18
Extensive	Mean	46.27	52.43	40.00	46.75
	Standard Deviation	9.69	10.07	7.55	5.80
	Median	45.90	50.00	39.30	45.00
	Range	46.00	47.00	36.00	23.00
	Median Deviation	8.12	7.83	6.26	4.63
Restricted	Media	50.18	56.01	43.37	45.98
	Standard Deviation	11.24	9.91	5.48	4.79
	Median	50.15	56.00	44.25	45.00
	Range	42.00	37.00	26.00	20.00
	Median Deviation	9.39	8.36	3.95	4.04

**Table 2:** Descriptive analysis of players’ perceptions regarding the intervention.

		Male (N = 14)		Female (N = 12)	
Dimensions	Category	Frequency	Percentage	Frequency	Percentage
Jump Improving	Yes	10	71.43	8	66.67
	No	1	7.14	2	16.67
	Not know	3	21.43	2	16.67
It is motivating to know the speed	Yes	14	100.00	12	100.00
	No	0	0.00	0	0.00
They learned to calculate the speed of the spike	Yes	2	14.29	1	8.33
	Yes (begin)	3	21.43	3	25.00
	Yes (middle)	2	14.29	1	8.33
	Yes (end)	1	7.14	1	8.33
	No	6	42.86	6	50.00
Improvement in the level of play during matches	Yes	7	50.00	8	66.67
	“Yes & no”	1	7.14	0	0.00
	No	3	21.43	3	25.00
	Not know	3	21.43	1	8.33

## Data Availability

The data that support the findings of this study are available from the corresponding author, E.O.-T., upon reasonable request. The data are not publicly available due to privacy and ethical restrictions.
